# Nontyphi Salmonella Empyema with Bronchopleural Fistula in a Patient with Human Immunodeficiency Virus

**DOI:** 10.1155/2018/4761725

**Published:** 2018-06-12

**Authors:** Douglas Bretzing, Tasnim Lat, Andrew Shakespeare, Mary Lee, Salim Surani, Shekhar Ghamande

**Affiliations:** ^1^Scott & White Healthcare and Texas A&M Health Science Center, College of Medicine, Texas, USA; ^2^Texas A&M Health Science Center, College of Medicine, Texas, USA

## Abstract

Patients with human immunodeficiency virus (HIV) have an increased risk of inoculation with nontyphoid* Salmonella* compared to the general population. While nontyphoid* Salmonella* commonly manifests as gastroenteritis,* Salmonella* bacteremia can be seen in patients with HIV. We present a case of disseminated* Salmonellosis* in a patient with HIV complicated by bronchopleural fistula and secondary empyema.* Case Presentation.* A 40-year-old African American male with HIV noncompliant with HAART therapy presented with complaints of generalized weakness, weight loss, cough, night sweats, and nonbloody, watery diarrhea of four weeks' duration. A computed tomography (CT) scan demonstrated a bilobed large, thick-walled cavitary lesion in the right upper lobe communicating with the pleural space to form a bronchopleural fistula. Thoracentesis yielded growth of nontyphi* Salmonella* species consistent with empyema; he was treated with intravenous Ceftriaxone and underwent placement of chest tube for drainage of empyema with instillation of alteplase/dornase twice daily for three days. Repeat CT chest showed a hydropneumothorax. The patient subsequently underwent video-assisted thoracoscopy with decortication. The patient continued to improve and follow-up CT chest demonstrated improved loculated right pneumothorax with resolution of the right bronchopleural fistula and resolution of the cavitary lesions.* Discussion.* We describe one of the few cases of development of bronchopulmonary fistula and the formation of empyema in the setting of disseminated* Salmonella*. Empyema complicated by bronchopulmonary fistula likely led to failure of intrapleural fibrinolytic therapy and the patient ultimately required decortication in addition to antibiotics. While* Salmonella* bacteremia can be seen in immunocompromised patients, extraintestinal manifestations of* Salmonella* infection such as empyema and bronchopleural fistulas are uncommon. Bronchopleural fistulas most commonly occur as a postoperative complication of pulmonary resection.* Conclusions*. This case highlights the unusual pulmonary manifestations that can occur due to disseminated* Salmonella* in an immunocompromised patient as well as complex management decisions related to these complications.

## 1. Introduction

Males afflicted with acquired immunodeficiency syndrome (AIDS) have an increased annual incidence of nontyphoid salmonella infections compared to the general male population [1–5]. Gastroenteritis accounts for approximately 70% of manifestation of infection with* Salmonella*, with extraintestinal manifestations occurring less commonly [6, 7]. When localized, extraintestinal* Salmonella* infection does occur, often in the setting of* Salmonella* bacteremia [7]. We present a case of disseminated* Salmonellosis* complicated by bronchopleural fistula with secondary in a patient with AIDS.

## 2. Case Description

**Figure 1:**
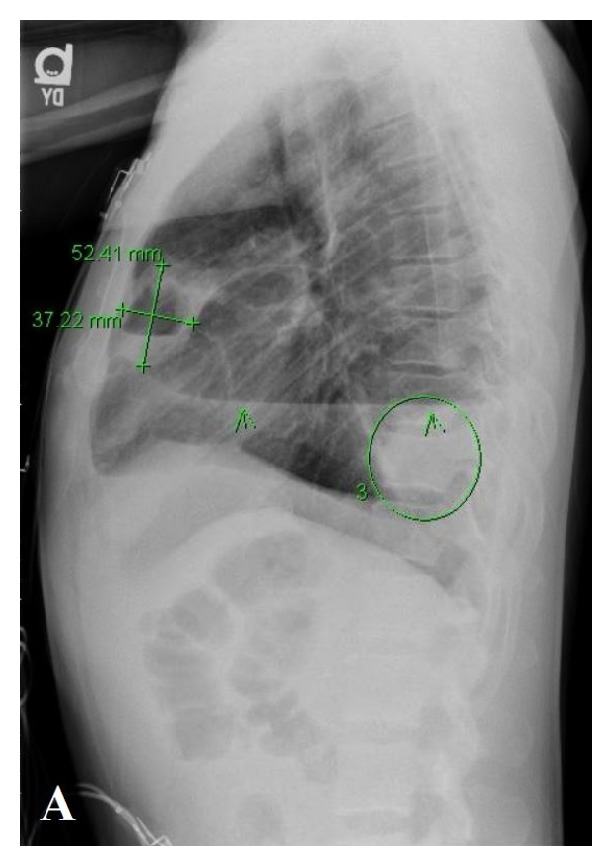
5.2 x 3.7 cm cavitary lesion containing an air-fluid level. An additional air-fluid level is present along the right lateral hemithorax.

**Figure 2:**
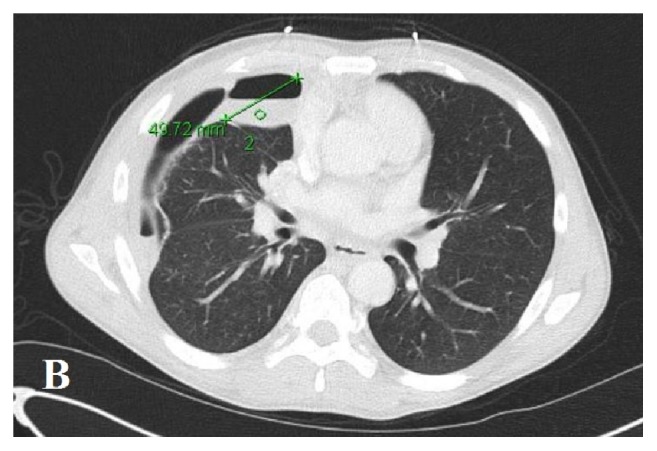
Hydropneumothorax.

**Figure 3:**
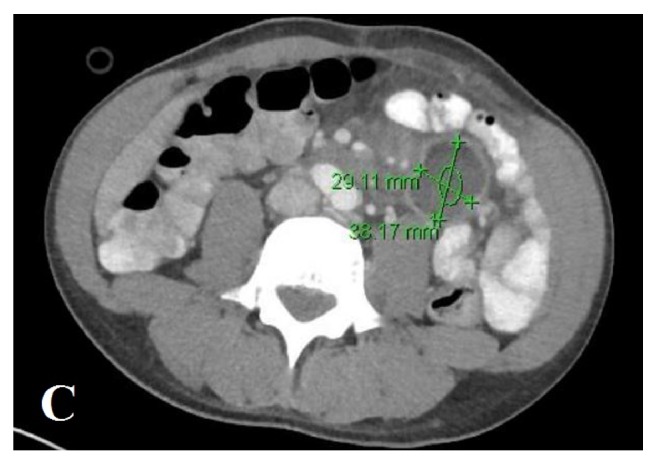
Two rim-enhancing hypodense lesions within the central mesentery of the abdomen.

**Figure 4:**
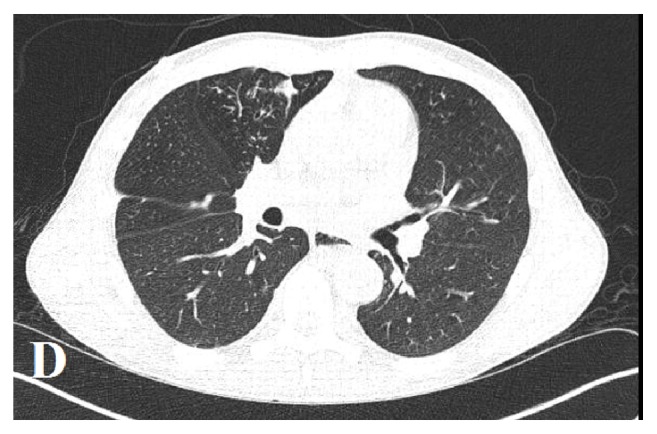
Resolution of hydropneumothorax.

A 40-year-old African American male presented with complaints of generalized weakness, unintentional weight loss (60 pounds over one-month period), cough, night sweats, and nonbloody, watery diarrhea of approximately four weeks' duration. The patient's medical history was additionally significant for hypertension and polysubstance abuse including tobacco (10 pack/year smoking history) and marijuana. He reported prior history of incarceration. He denied recent travel or animal exposures at home. He resided with his mother, for whom he was the primary caregiver. He denied having sexual activity within the past 6 months. Initial vitals in the emergency department were significant for tachycardia with HR in the 120's. Physical examination at the time of admission revealed a thin, nontoxic appearing male. Cardiac exam revealed tachycardia, with no murmurs or rub. Lung exam revealed decreased breath sounds in the bilateral lower lung fields with tubulovesicular sound emanating from right upper lung field. His abdomen was soft and nontender. He had no focal neurologic deficits. Initial laboratory workup was significant for absolute CD4 count of 26 (3%). Urinalysis showed cloudy urine with 1+ blood, positive nitrite, 3+ leukocyte esterase, WBC >50/HPF, RBC 3-9/HPF, and many bacteria. Chest X-ray (CXR) (PA and lateral views) in the emergency department revealed a cavitary lesion with an air-fluid level within the anterior medial right hemithorax and a loculated hydropneumothorax along the right lateral lung base (Figure 1). CT chest with contrast demonstrated two large, thick-walled cavitary lesions originating within the right lung parenchyma that appeared to communicate. The smaller lesion measured up to 5 cm and the larger lesion contained an air-fluid level. This was interpreted as demonstrating a complex bronchopleural fistula and associated empyema (Figure 2). His treatment was initiated with intravenous Ceftriaxone and Metronidazole. The patient's stool PCR isolated a* Salmonella* species. Diagnostic thoracentesis yielded purulent fluid with WBC 505,000 (73% segmented neutrophils, 8% lymphocytes, and 19% macrophages), RBC 0, pH 6.0, protein 3.7, LDH 41,239, and glucose 12. Pleural fluid culture was positive for* Salmonella* species. Due to presence of empyema, a right-sided chest tube was placed followed by instillation of tissue plasminogen activator (r-tPA) and DNase twice daily for three consecutive days. His urine culture yielded nontyphi* Salmonella*. CT abdomen and pelvis with contrast demonstrated two rim-enhancing hypodense lesions (measuring 2.9 x 3.8 cm transverse and 3.2 x 4.5 cm transverse) within the central mesentery of the abdomen (Figure 3). Intra-abdominal drains were placed under CT guidance into the mesenteric abscesses, from which nontyphi* Salmonella* eventually grew late in hospital course. Due to immunocompromised status with cavitary lesions on imaging, acid fast bacilli (AFB) smears were obtained along with interferon gamma release assay which were negative. On hospital day #4 the patient's right-sided chest tube was noted to have persistent air leak. CT chest without contrast confirmed persistent right-sided hydropneumothorax with centrilobular ground glass opacities with lung entrapment. On hospital day #10, the patient underwent right video-assisted thoracoscopic surgery (VATS) with decortication. Pleural peel pathology revealed pleural fibrosis, focal chronic inflammation, and mild anthracosis. The patient completed an additional two weeks of antibiotic therapy with intravenous Ceftriaxone from the date of decortication. On follow-up at four-months, CT chest demonstrated improved but persistent loculated right pneumothorax with resolution of the right lung cavitary lesions. At seven months, the CT demonstrated complete resolution of the right-sided pneumothorax (Figure 4).

## 3. Discussion

We described a case of bronchopleural (BP) fistula with empyema caused by disseminated* Salmonellosis* in a patient with AIDS who was noncompliant with HAART therapy.* Salmonella* causing empyema in HIV patients is rarely described. Borge* et al.* described a cohort of HIV infected patients with empyema, with the most common bacteria being* Staphylococcus aureus* and gram negative bacilli. However,* Salmonella* was not reported in any of those patients. Six out of 23 patients had a BP fistula, none of whom required surgery, although the length of stay was increased [8]. These patients tended to have bacteremia and polymicrobial flora. 18 out of 23 were managed with tube thoracostomy for an average of 14 ±41 days. The other patients did not require a chest tube [8].

BP fistula occurrence in AIDS patients is described in literature most commonly due to* Pneumocystis jiroveci* [9–11]. Our patient's empyema was treated with intrapleural r-tPA and DNnase which has been shown to be effective for empyema but nonetheless failed to resolve empyema in our patient's case [12]. We hypothesize that bronchopleural fistula contributed to treatment failure with intrapleural tPA and dornase. In a cohort of 27 AIDS patients with pneumothorax (PTX), 10 had a bronchopleural fistula [11]. Of all the patients with PTX, 18 out of 44 patients received tube thoracostomy alone (41%), tube thoracostomy with pleurodesis in 2/8 PTXs (25%), and thoracotomy with bleb resection and pleurodesis in 1/3 PTXs (33%) were used. Eight out of 10 patients with BP fistula were discharged home on a Heimlich valve [11]. Wait and coworkers studied the patients undergoing thrombolytics versus VATS. Their study favored the VATS with 91% success rate versus 44% success rate with the streptokinase group [13]. Piccolo et al. studied the use of intrapleural tPA and deoxyribonuclease therapy for the treatment of empyema. They found this combination therapy to provide cure in over 90% of patients without the surgery [14].

Our patient ultimately required VATS decortication for resolution of his right-sided empyema and bronchopleural fistula. We suspect that our patient required VATS decortication for resolution of his empyema secondary to development of lung entrapment and bronchopleural fistula. Surgical treatment of empyema in HIV patients has a reasonable success rate. Khwaja* et al.* described a complication rate of 28.5% with no immediate postoperative mortality in a series of 21 patients. Those with CD4 counts of < 200 cells/*μ*l were more likely to require resection and had a longer length of stay [15]. Chronic, organizing empyema can require open decortication due to the presence of a thick visceral pleural peel complicating VATS decortication; however, VATS exploration could be considered as a reasonable initial approach [16]. In this case, lysis of the dense, pleural adhesions, and resection of the pleural peel were successfully accomplished via VATS decortication.

## 4. Conclusion

This case illustrates unique presentation of disseminated* Salmonellosis* in a patient with AIDS who was noncompliant with HAART therapy. It also demonstrates several complications of pneumonia, including empyema, bronchopleural fistula, and lung entrapment. Our approach to treatment of these complications using intrapleural fibrinolytics and VATS decortication, in addition to antibiotics and chest tube placement, is outlined.
